# Future Objectivity Requires Perspective and Forward Combinatorial Meta-Analyses

**DOI:** 10.3389/fpsyg.2022.908311

**Published:** 2022-06-17

**Authors:** Barbara Hanfstingl

**Affiliations:** Institute for School and Instructional Development, University of Klagenfurt, Klagenfurt, Austria

**Keywords:** objectivity, perspective, subjectivity, specification curve analysis, meta–meta-analyses, combinatorial meta-analysis

## Abstract

This manuscript contributes to a future definition of objectivity by bringing together recent statements in epistemology and methodology. It outlines how improved objectivity can be achieved by systematically incorporating multiple perspectives, thereby improving the validity of science. The more result-biasing perspectives are known, the more a phenomenon of interest can be disentangled from these perspectives. Approaches that call for the integration of perspective into objectivity at the epistemological level or that systematically incorporate different perspectives at the statistical level already exist and are brought together in the manuscript. Recent developments in research methodology, such as transparency, reproducibility of research processes, pre-registration of studies, or free access to raw data, analysis strategies, and syntax, promote the explication of perspectives because they make the entire research process visible. How the explication of perspectives can be done practically is outlined in the manuscript. As a result, future research programs can be organized in such a way that meta-analyses and meta-meta-analyses can be conducted not only backward but forward and prospectively as a regular and thus well-prepared part of objectification and validation processes.

## Introduction

Objectivity is a core criterion for achieving sound scientific results. It reflects a central specificity of modern science. The concept bears different, although related, definitions. According to Gaukroger (2012), objectivity is the capacity to stand back from our perceptions, beliefs, and opinions, and to shift perspective. From an anthropological perspective, Tomasello (2020) sees the need for social inclusion already in children, which requires objectivity as a developmental prerequisite for adopting different perspectives and social inclusion. The American Psychological Association (APA)^1^ defines objectivity as (1) “the tendency to base judgments and interpretations on external data rather than on subjective factors, such as personal feelings, beliefs, and experiences; (2) a quality of a research study such that its hypotheses, choices of variables studied, measurements, techniques of control, and observations are as free from bias as possible;” and as opposite of subjectivity. In test statistics, objectivity is one of the three main quality criteria for psychological tests, along with reliability and validity, and refers to the test’s procedure, result, analysis, and interpretation, being independent of the person conducting the test.

The modern idea of objectivity grew in times of Enlightenment when scientific thinking took over the dogmatic thinking of the Christian church in Europe. According to Daston and Galison (2010), who provide a comprehensive history of the concept, objectivity, as we use it in science today, emerged in the mid-nineteenth century and is associated with the ability to display “the world as it is,” aided by the invention of photography. From a traditional scientific view, Popper (1972) saw objectivity as the correct application of scientific methods and procedures to make accurate predictions. This view on objectivity comes very close to what recent methodologies understand as objectivity: findings are scientific if they are reproducible and intersubjectively testable. Scientific thinking deals with objective facts; if knowledge is scientific, it is objective and objectifiable. Ideally, objectivity ensures the necessary distance to the subject of interest, it prevents the scientist from subjectivity and (emotional) involvement.

Scientific communities agree that objectivity is necessary to ensure that a scientific fact is indeed a scientific fact. Nevertheless, several authors from different fields question the current concept of scientific objectivity as a sufficient criterion for establishing a scientific fact. In the Stanford Encyclopedia of Philosophy, Reiss and Sprenger (2020) question the reachability of objectivity and see the final understanding of it as an ongoing project. As early as 1933, Rosenthal and Rosnow (2009; reprint) point out in their research the influences of human bias – subjectivity, to put it succinctly – on research findings. Recent state-of-the-art publications show that human bias significantly affects scientific results, even when we strive for objectivity in testing the same hypotheses and use accepted scientific methods to test them (e.g., Silberzahn et al., 2018; Bastiaansen et al., 2020; Schweinsberg et al., 2021). These studies show that the current state of the methodology does not meet objectivity, as researchers must make individual decisions and specifications on how to conduct a study. Therefore, one reason we struggle with objectivity lies in the historical and social developments of the so-called postmodern era. Both the world and science are realized as increasingly complex, interconnected, and systemic.

Undoubtedly, because psychological phenomena have traditionally been considered unobservable, there is reason to believe that objectivity may be impossible to achieve. Further, in psychology, mechanical objectivity works for non-complex matters such as the Weber-Fechner law, but more complex psychological theories apparently lack objectivity. This could be one reason why psychology as a discipline suffers most from the replication crisis, as Freese and Peterson (2018) note. The troubleshooting process is intense, but also urgent. The more latent constructs, statistical sophistication, and implicit probability calculations entered the methodological logic of psychological science, and the more theoretical (Fiedler et al., 2021), metrological (Uher, 2018, 2020, 2021), contextual (Borgstede and Scholz, 2021), and epistemological (Meehl, 2009; Hanfstingl, 2019) considerations were ignored, the greater the problem became (e.g., Open Science Collaboration, 2015).

## Considering “Perspective”

The term “perspective” has a tradition in psychological sciences, but less on an epistemological level than from a cognitive-developmental psychological perspective. The APA (see text footnote 1) defines perspective as “(1) the ability to view objects, events, and ideas in realistic proportions and relationships; (2) the ability to interpret relative position, size, and distance of objects in a plane surface as if they were three-dimensional; (3) the capacity of an individual to take into account and potentially understand the perceptions, attitudes, or behaviors of themselves and other individuals; and (4) a particular way of looking at events or situations: a stance or philosophical position.”

In recent contributions to the philosophy and sociology of science, regarding the objectivity problem, the idea of perspective is discussed epistemologically. For example, Susen (2015) describes the opposition of “perspective versus truth” as an essential criterion of the so-called “postmodern turn” in the social sciences. He argues that perspective could replace the binary concept of objectively true and objectively false in science. Additionally, from a postmodern, feminist, and standpoint tradition, Harding (1995, 2015) suggests using the term “strong objectivity,” which means considering traditional scientific objectivity and the perspective of the scientist who achieves a scientific result. Similarly, Daston (1992) speaks of a perspectival objectivity. Tannoch-Bland (1997) claims moral objectivity since morality is often claimed by authorities, depending on historical contexts. These considerations are in line with the ideas by Daston and Galison (2010),Gaukroger (2012), and Tomasello (2020). Epistemologically, there is an agreement that we must not abandon the idea of objectivity, but we have to enrich the original idea with perspective-taking. However, we require a formalized solution, which can be realized on a methodological, empirical, and statistical level.

## Collective Objectivity in a Statistical Understanding

For Freese and Peterson (2018), a collective level of objectivity is the only way to escape individual perspectives and subjectivity, and they suggest using meta-analyses to address this issue. The authors call this approach collective or statistical objectivity, seeing meta-analyses as the apex of objectivity (Freese and Peterson, 2018). At first glance, meta-analyses meet the criteria of combining different single studies, therefore different perspectives. It is no coincidence that they are hyped in the current scientific milieu (e.g., Iliescu et al., 2022). Freese and Peterson (2018) argue that single study results often are influenced by scientists’ “scientific selves,” which, in turn, are affected by different interests, such as emotional or economic. From a cognitive angle, Hanfstingl (2019) mentions the scientific-selves-biases in the work with latent constructs, emphasizing that these problems are grounded to a certain degree on our cognitive automatisms. Meta-analyses, unlike single studies, can reveal statistical effects that would otherwise go undetected.

However, researchers agree that meta-analyses only provide objective knowledge when they are informed by modern quality criteria, such as standardized reporting guidelines and free access to all data and analysis syntaxes, which is often not the case for meta-analyses (Lakens et al., 2016; Polanin et al., 2020). Glass (2015) acknowledges that the initial phase of meta-analyses was characterized by arbitrary decisions and a lack of quality criteria. Still, the provided information is not sufficient to maintain reproducibility and, therefore, higher objectivity (Maassen et al., 2020). Several authors propose clear criteria of reproducibility for future science (e.g., Gurevitch et al., 2018) without considering different perspectives to reach objectivity. Munafò et al. (2017) point to perspective-taking by mentioning team science and the advantages of collaboration, but not systematically and in-depth. However, there are several proposed solutions that have already been published.

**FIGURE 1:**
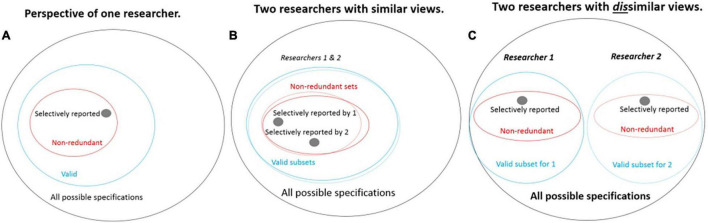
Sets of possible specifications as perceived by researchers. **(A)** The set of specifications reported in an article are a small subset of those the researcher would consider valid to report. **(B)** Different researchers may have similar views on the set of valid specifications but report quite different subsets of them. **(C)** Different researchers may also disagree on the set of specifications they consider valid (Simonsohn et al., 2020, p. 2, Figure 1; reprinted with permission by the first author).

**FIGURE 2:**
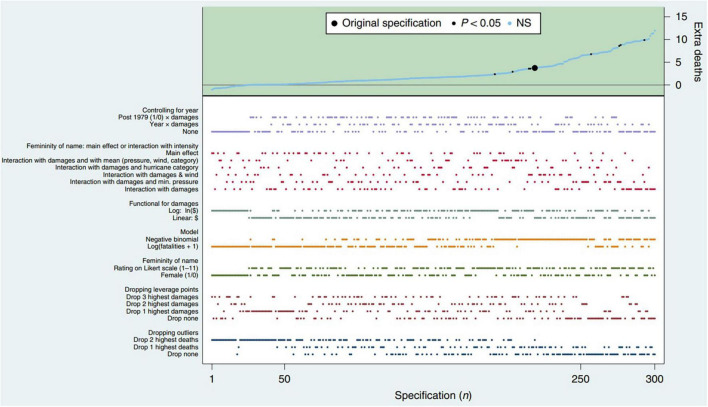
Descriptive specification curve. Each dot in the top panel (green area) depicts the marginal effect, estimated at sample means, of a hurricane having a female rather than male name; the dots vertically aligned below (white area) indicate the analytical decisions behind those estimates. A total of 1,728 specifications were estimated; to facilitate visual inspection, the figure depicts the 50 highest and lowest point estimates and a random subset of 200 additional ones, but the inferential statistics for specification curve analysis include all 1,728 specifications. NS, not significant (Simonsohn et al., 2020, p. 3, Figure 2; reprinted with permission by the first author).

Voracek et al. (2019) point to approaches that are able to illustrate researchers’ degrees of freedom systematically. For this, they combine solutions developed by Olkin et al. (2012),Steegen et al. (2016), and Simonsohn et al. (2020). Simonsohn et al. (2020) developed their approach for single studies and called it the Specification Curve Analysis, which aims to specify all reasonable and arguable decisions and specifications to answer a research question. The authors also incorporate the problem of different scientists’ perspectives (Figure 1) and aim to systematically depict them on a “specification curve.” These specifications have to (1) sensibly test the research question, (2) be expected to be statistically valid, and (3) not be redundant with other specifications in the set (Simonsohn et al., 2020). The specification curve (Figure 2) describes the estimated effect sizes across all specifications, organized around a dashboard chart showing the operationalizations behind each result. Thus, an estimation of the factors influencing the results (decisions on theoretical and methodological approaches, interpretation habits, i.e., scientific selves), can be illustrated in a structured and comprehensible way on the basis of many single studies. Steegen et al. (2016) provide the idea of a multiverse analysis as a similar approach using additional plot alternatives.

Because Steegen et al.’s (2016) and Simonsohn et al.’s (2020) approaches only focus on the single-study-level, Voracek et al. (2019) combine specification curves with an all-subsets combinatorial meta-analysis approach by Olkin et al. (2012). In other words, specification curves combined with combinatorial meta-analyses lead to a systematic overview of possible outcomes resulting from various decisions made by scientists at a meta-meta level. However, Voracek et al. (2019) mention two main problems in their approach. First, the analyses quickly become unfeasible due to the many possible combinations, which could be met with a specific bootstrap strategy they suggest in their manuscript. Second, the approach is still not free of subjective considerations, as “factors need to be tailor-made each time anew, informed by specific debates in the primary literature or by prior related meta-analyses” (Voracek et al., 2019, p. 78). Although researchers get a completely new meta-level of knowledge with this approach family, it seems that the last decision level stays human-biased and perspective-dependent. Nonetheless, this analysis strategy allows many human biases to be made explicitly visible at an individual studies level and meta-analyses in an unprecedented, systematic way. This has been shown by two recent applications of these approaches, both on a meta-meta-level: Dürlinger and Pietschnig’s (2022) investigation of the association between intelligence and religiosity, and Vilsmeier et al.’s (2021) analysis of the stability of birth order effects.

## Conclusion

No scientific method ensures objectivity automatically, and mechanical objectivity is hard to meet for many scientific results. There is a high agreement that objectivity has to be redefined formally. Most authors working on the objectivity problem suggest including perspectives into the concept of objectivity. For example, Harding (2015) says that objectivity is weak as long as we do not consider perspectives; Tannoch-Bland (1997) focuses on moral objectivity when considering authorities’ perspectives, both from a feminist context; Susen (2015) contrasts perspective with truth within a postmodern turn, and authors who offer statistical and computational solutions include researchers’ degrees of freedom, that is, their perspectives (Olkin et al., 2012; Steegen et al., 2016; Freese and Peterson, 2018; Voracek et al., 2019; Simonsohn et al., 2020). There is also an agreement that, ultimately, influences of human biases remain also on a meta-meta level.

However, is it possible to avoid the frequently-mentioned postmodern arbitraries? I would say, yes, more than that. If we systematically consider objectivity, including diverse perspectives, the validity of science grows instead of shrinks. The more result-biasing specifications and perspectives are known, the more a phenomenon of interest can be disentangled from them. This assumption is supported by recent developments in (psychological) science, in which a major goal is to conduct research in the vein of, for example, an open science policy that can be applied at both single-study and meta-study levels. Many rules were brought together by the open science movement, like ensuring transparency and reproducibility of research processes, preregistrations of studies, or open access to raw data, analysis strategies, syntaxes, and manuscripts. Several older ideas are consistent with systematically accounting for different contextual influences when, for example, randomization tests are used in smaller data sets. Dugard et al. (2012) integrate this idea at the planning stage of a research design, which already implies a prospective validation approach and an orientation toward preregistration, respectively. There is an agreement that it is barely possible to avoid the degrees of freedom when deciding how to frame a study or meta-study. However, as one of the reviewers of this manuscript mentioned, researchers’ profound subject matter knowledge helps to use these degrees of freedom in the interest of scientific progress, which goes in line with the argumentation of Hanfstingl (2019).

Open access to research at all its stages opens up the possibility of organizing research programs in such a way that meta-analyses and meta-meta-analyses can be conducted not only backward but forward and prospectively as a regular and thus well-prepared part of objectification and validation processes. Although open access is not necessarily a prerequisite for this consideration, it does bundle together ideas for increasing the objectivity and validity of scientific results. Initiatives, such as big team programs, foster such research strategies and are growing in different fields, be it in general medical science (Steer et al., 2017), psychological science (Forscher et al., 2020), or in a more specific manner, like addiction science (e.g., Pennington et al., 2022). As mentioned above, the troubleshooting process is urgent, but also intense.

## Author Contributions

BH was the sole author of the manuscript and included the conception and organization of the ideas in the manuscript, recherche of the references, revised, and approved the submitted version.

## Conflict of Interest

The author declares that the research was conducted in the absence of any commercial or financial relationships that could be construed as a potential conflict of interest.

## Publisher’s Note

All claims expressed in this article are solely those of the authors and do not necessarily represent those of their affiliated organizations, or those of the publisher, the editors and the reviewers. Any product that may be evaluated in this article, or claim that may be made by its manufacturer, is not guaranteed or endorsed by the publisher.

## Footnotes

1
https://dictionary.apa.org/
